# Phytochemical Constituents and Biological Activity of Wild and Cultivated *Rosmarinus officinalis* Hydroalcoholic Extracts

**DOI:** 10.3390/antiox12081633

**Published:** 2023-08-18

**Authors:** Rosaria Francolino, Mara Martino, Lucia Caputo, Giuseppe Amato, Giuseppina Chianese, Ernesto Gargiulo, Carmen Formisano, Benedetta Romano, Giuseppe Ercolano, Angela Ianaro, Laura De Martino, Vincenzo De Feo

**Affiliations:** 1 Department of Pharmacy, University of Salerno, Via Giovanni Paolo II, 132, 84084 Fisciano, Italy; rfrancolino@unisa.it (R.F.); martinomara966@gmail.com (M.M.); lcaputo@unisa.it (L.C.); gamato@unisa.it (G.A.); defeo@unisa.it (V.D.F.); 2 Department of Pharmacy, School of Medicine and Surgery, University of Napoli Federico II, Via D. Montesano, 49, 80131 Napoli, Italy; g.chianese@unina.it (G.C.); ernesto.gargiulo@unina.it (E.G.); benedetta.romano@unina.it (B.R.); giuseppe.ercolano@unina.it (G.E.); angela.ianaro@unina.it (A.I.); 3 Institute of Food Science, National Research Council (C.N.R.), Via Roma, n. 60, 83100 Avellino, Italy

**Keywords:** *Rosmarinus officinalis*, hydroalcoholic extract, antioxidant activity, chemical composition, reactive oxygen species, anti-inflammatory activity

## Abstract

*Rosmarinus officinalis* L. is an aromatic evergreen plant from the Lamiaceae family. The purpose of this study was to compare the chemical profile and bioactivities of hydroalcoholic extracts derived from wild and cultivated *R. officinalis*. The chemical composition of the extracts was evaluated via LC–MS analysis, which revealed the presence of a wide range of phenolic compounds, including flavonoids, phenolic and terpenes. Both extracts showed a similar interesting antioxidant activity, probably related to their content of phenol and flavonoids. The analysis of anti-acetylcholinesterase (AChE), anti-butyrylcholinesterase (BChE), and anti-α-amylase activities showed analogous inhibition, except for AChE, in which the wild type was more active than the cultivated one. Finally, in vitro studies were performed using the J774A.1 murine macrophage cell line, to characterize the anti-inflammatory and the antioxidant effects of the extracts. As expected, pretreatment with the extracts significantly reduced the production proinflammatory cytokines and ROS through modulation of the nitric oxide pathway and the mitochondrial activity. Importantly, it is observed that the anti-inflammatory effect of the extracts was explicated through the inhibition of NF-kB and its downstream mediator COX-2. Collectively, these results demonstrated that these extracts could represent a starting point for developing novel therapeutic strategies for the treatment of inflammation-based diseases. Moreover, since no significant changes were observed in terms of composition and activity, both wild and cultivated *R. officinalis* extracts can be recommended for food and pharmaceutical purposes.

## 1. Introduction

*Rosmarinus officinalis* L., rosemary, is an evergreen aromatic plant from the Lamiaceae family. Rosemary is a shrub with a powerful pungent aroma, is 1–2 mt tall, and has deep and resistant roots. The leaves are dark green and elongated, and the flowers are white or purple. *R. officinalis* is cultivated worldwide but is native to the Mediterranean area: in these areas, there are numerous cultivated and wild forms. Rosemary fresh leaves are used in the Mediterranean diet as flavorings and spices; leaf extracts have been reported for preventing food deterioration [1].

*R. officinalis* has multiple bioactivities including antioxidant, antibacterial, hypoglycemic, anticancer, hepatoprotective, anti-inflammatory, and antithrombotic activities [2,3]. The biological activities of rosemary extracts are mostly attributed to the presence of polyphenols, such as rosmarinic acid, and phenolic diterpenes, such as carnosic acid and carnosol [4,5]. In particular, the presence of polyphenols affects its antioxidant activity. Rosmarinic acid, carnosic acid, carnosol, rosmadial, and genkwanin are responsible for its anti-radical activity [6]. The antioxidant activity of these compounds is closely related to other bioactivities, such as cytoprotective and anticancer, with these compounds being capable of neutralizing reactive oxygen species (ROS) [7]. Moreover, previous studies have shown that polyphenols exert an inhibiting action on α-amylase enzyme, which catalyzes the first step of starch digestion [8].

Different rosemary extracts also showed anti-acetylcholinesterase (AChE) activity and play an important role in some neurodegenerative ailments such as Alzheimer’s disease, dementia, and Parkinson’s disease [9]. Moreover, plasma and/or tissue levels of several enzymes such as acetylcholinesterase, butyrylcholinesterase, and amylase can be considered as biochemical markers to discover and diagnose the existence of acute, chronic, and low-grade systemic inflammation [10,11,12].

The different biological activities of *R. officinalis* extracts cannot be attributed to a single class of substances but, probably, to a synergistic action between some bioactive components [13]. Moreover, the percentage of secondary metabolites could depend on various factors [13,14]. Changes to the plant environment, such as transplantation from a wild habitat to a cultivated field, can produce modifications in plant development, biochemistry, and the quantity of active substances [15]. Cultivated material is sometimes believed to miss the ‘power’ of wild medicinal plants [16], although this concept is not supported by evidence. The cultivation of officinal plants could be a solution to the uncontrolled wild harvesting that affects medicinal species. Furthermore, the advantage of a cultivation could be that of maintaining a continuous and controlled supply for possible production.

The goals of this study were to compare (i) the chemical composition of the hydroalcoholic extract from wild (WRO) and cultivated (CRO) *Rosmarinus officinalis* using LC-MS; (ii) their total polyphenol content (TPC), total flavonoid content (TFC), and antioxidant activities using DPPH (1,1-diphenyl-2-picrylidrazyl) and FRAP (ferric-reducing antioxidant power) assays; and (iii) the possible anti-acetylcholinesterase (AChE), anti-butyrylcholinesterase (BChE), and anti-α-amylase activities of the extracts. In addition, the anti-inflammatory effects of WRO and CRO were further characterized through an in vitro approach using J774 murine macrophages. In particular, the ability of both CRO and WRO to modulate M1-polarized murine macrophage functions was evaluated. Thus, their ability to modulate ROS and proinflammatory cytokine production through qPCR and a flow cytometry analysis was assessed.

## 2. Materials and Methods

### 2.1. Reagents

The reagents included 2,2-difenil-1-picrylidrazyl (DPPH), 2,4,6 triperidol-s-triazine (TPTZ), 5,5′-dithiobis(2-nitrobenzoic acid) (DTNB), 6-hydroxy-2,5,7,8-tetramethylchroman-2-carboxylic acid (Trolox), acarbose, acetylcholinesterase (AChE), acetylthiocholine iodide (AChI), aluminum chloride, ascorbic acid, butyrylcholinesterase (BChE), dinitrosalicylic acid (DNSA), ferric chloride, ferrous sulfate heptahydrate, Folin–Ciocalteu′s phenol reagent, gallic acid, hydrochloric acid, porcine pancreatic α-amylase (PPA), potassium sodium tartrate, quercetin, sodium acetate, sodium bicarbonate, sodium hydroxide, sodium nitrite, sodium tartrate, and soluble starch.

All used reagents were purchased from Sigma Aldrich (Milan, Italy). The solvents (analytical- and deuterated-grade)—formic acid, water, acetonitrile and methanol—were purchased from Sigma Aldrich (Milan, Italy).

### 2.2. Plant Material

*R. officinalis* L. was collected in the Campania region: the cultivated plant was supplied by a company located in Pontecagnano (SA) (40°39′ N 14°53′ E, 28 m.a.s.l.), and the wild plant was collected in the same area. The cultivation conditions were reported as follows: temperate climate, with warm summers and moderate dry winters; open field fertigation with mulch. A voucher specimen of each sample was stored in the Herbarium of the Medical Botany Chair, Department of Pharmacy, Salerno University.

### 2.3. Extract Preparation of R. officinalis L.

The aerial parts of the plants were cleaned and left to air dry. The dried plants, cut into small pieces, were macerated with EtOH 70% (*v*/*v*). The extraction was repeated three times, changing the solvent to increase the extract quantities. The extracts were filtered using paper filters, frozen, and subjected to freeze-drying to obtain the dried extracts. The hydroalcoholic extracts resulted in 7% and 10% yields, respectively, for WRO and CRO. Samples were stored at room temperature until further analysis.

### 2.4. Chemical Analysis: LC-HRESIMS/MS Analysis

The LC-HRESIMS/MS analyses of the hydroalcoholic extracts were performed via high HPLC-MS analysis using a Thermo LTQ Orbitrap XL mass spectrometer (Thermo Fisher Scientific Spa, Rodano, Italy) equipped with electrospray ion (ESI) MAX source coupled to a Thermo U3000 HPLC system (Agilent Technology, Cernusco sul Naviglio, Italy). Chromatographic separation was achieved using a Kinetex Polar C18 column (100 × 3.0 mm, 100 Å, 2.6 µm). The injection volume was 5 µL, the flow rate was 0.5 mL/min, and the mobile phase consisted of a combination of A (0.1% formic acid in water, *v*/*v*) and B (MeCN). Chromatographic analyses were conducted using a linear gradient from 5% to 95% B in 15 min and held at 95% B for 5 min. The HRMS and MSn spectra, in the positive mode, were recorded in data-dependent acquisition mode, inducing fragmentation of the five most intense peaks for each scan. The source conditions were as follows: spray voltage, 4.8 kV; capillary voltage, 31 V; auxiliary gas, 15 (arbitrary units); sheath gas, 32; capillary temperature, 285 °C; normalized collision energy, 30; isolation width, 2.0; activation Q, 0.250; and activation time, 30 ms. The acquisition range was *m*/*z* 150−1500.

### 2.5. Chromatographic Purification of Compound **4**

A sample of CRO and WRO (15 mg each) was separated via analytical RP-18 HPLC performed on an Agilent instrument, using a Synergi Polar-RP, 4μm 250 mm × 4.6 mm column. The mobile phase was a mixture of A, water−formic acid (99.9:0.1, *v*/*v*), B, and acetonitrile, with a gradient programmed as follows: starting conditions, A 95% B 5%; 5–8 min, A 85% B 15%; 11–15 min, A 75% B 25%; 30–35 min, A 50% B 50%; and 40–45 min, A 5% B 95%. The injected volume was 100 μL, and the flow rate was 1 mL/min. The UV detection wavelength was set at 275 nm. This separation afforded compound **4**, identified via NMR as luteolin-3′-*O*-(3″-*O*-acetyl)-β-D-glucuronide (0.5 mg, *t_R_* 23.6 min).

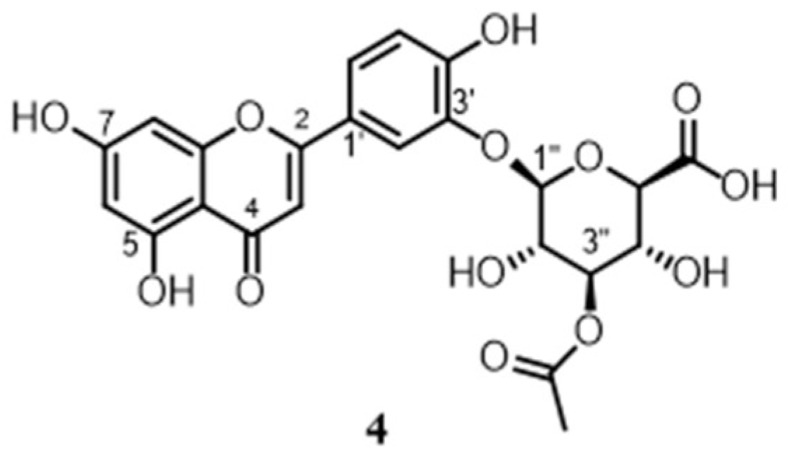


*Luteolin-3*′*-O-(3*″*-O-acetyl)-β-D-glucuronide* (**4**). Colorless amorphous solid. ^1^H NMR (methanol-*d*4, 700 MHz) *δ*_H_ 8.08 (1H, s, H-2′), 7.60 (1H, d, J = 8.2, H-6′), 6.98 (1H, d, J = 8.2, H-5′), 6.86 (1H, s, H-3), 6.61 (1H, s, H-8), 6.18 (1H, s, H-6), 5.11 (1H, t, J = 9.4, 18.8, H-3″), 4.96 (1H, d, J = 7.7, H-1″), 3.91 (1H, d, H-5″), 3.75 (1H, dd, H-2″), 3.73 (1H, dd, H-4″); 2.15 (3H, s, H-2‴); ^13^C NMR (methanol-*d*4, 175 MHz) *δ*_C_ 182.4 (C4), 169.7 (C6), 169.2 (C-1‴) 164.8 (C-7), 164.0 (C-2), 162.7 (C-5), 157.6 (C-9), 146.4 (C-3′), 122.7 (C-6′), 121.0 (C-1′), 116.9 (C-2′), 116.6 (C-5′), 103.7 (C-10), 103.5 (C-1″), 102.6 (C-3), 98.8 (C-6), 93.8 (C-8), 76.6 (C-3″), 75.6 (C-5″), 71.8 (C-4″), 70.4 (C-2″), 20.9 (C-2‴); HRESIMS (positive ions): *m*/*z* 505.0957 [M+H]^+^ (calcd for C_23_H_21_O_13_ 505.0988).

#### 2.5.1. Quantitative Analysis of Major Phenolic Compounds

The quantitative profiling of the hydroalcoholic extracts of *R. officinalis* was performed via high HPLC-UV analysis using an Agilent instrument equipped with a Phenomenex Kinetex Polar C18 column (100 × 3.0 mm, 100 Å, 2.6 µm). The chromatographic profile was obtained using the same chromatographic method used for the LC-HRESIMSMS analysis. The injection volume was 10 µL, and the UV detection wavelength was set at 280 nm.

The calibration curve was obtained using salvigenin as internal standard at concentrations of 125, 250, 500, 1000, 2000, and 4000 ppm. The coefficient determination (R^2^) of the calibration curve had good fitness (0.9975–0.99874); salvigenin present in the extracts was quantified and used as reference to relatively quantify the other components by means of the % Area.

#### 2.5.2. Analysis of Total Phenol and Flavonoid Content

Total phenolic content (TPC) in the rosemary extracts was measured spectrophotometrically according to the Folin–Ciocalteu method [17]. Firstly, 10 μL of the diluted extract or gallic acid standard solution, 790 μL of deionized water, and 50 μL of the Folin–Ciocalteu reagent were put in the cuvettes; after 8 min, 150 μL of Na_2_CO_3_ was also added. After 2 h of incubation, at room temperature, the absorption value of the mixture was detected at 765 nm, using a Thermo Scientific Multiskan GO spectrophotometer (Thermo Fischer Scientific, Vantaa, Finland). For determining the calibration curve, gallic acid was used as a standard. The total phenolic content was expressed as mg gallic acid equivalent (mgGAE)/g of hydroalcoholic extract.

Total flavonoid content (TFC) was measured spectrophotometrically via the aluminium chloride colorimetric method following the protocol of Baba and Malik [18] with some modifications. Firstly, 50 μL of the diluted extract or quercetin standard solution, and 30 μL of a 5% NaNO_2_ were put in the cuvettes; after 5 min, 30 μL of a 10% AlCl_3_ was also added. After a 5 min incubation, 200 μL of NaOH (1M) was added. Finally, water was added up to a volume of 1 mL. The absorption value of the mixture was detected at 510 nm using a Thermo Scientific Multiskan GO spectrophotometer (Thermo Fischer Scientific, Vantaa, Finland). For determining the calibration curve, quercetin was used as a standard, and the total flavonoid content was expressed as mg quercetin equivalent (mgQE)/g of hydroalcoholic extract.

### 2.6. Antioxidant Activity

#### 2.6.1. DPPH Assay

The antiradical activity was determined using the stable 1,1-diphenyl-2-picrylhydrazyl radical (DPPH) following the protocol of Brand-Williams [19] with some modifications reported by De Martino and coworkers [20].

#### 2.6.2. FRAP Assay

The FRAP assay (FRAP is an acronym for “Ferric Ion Reducing Antioxidant Power”) was performed following the protocol of Benzie and Strain [21].

A FRAP reagent is a solution consisting of 23 mM acetate buffer (pH 3.6), 10 mM of tripyridyl triazine (TPTZ) in 40 mM of HCl, and 20 mM of FeCl_3_ (in a 10:1:1 ratio). Different concentrations of ferrous sulfate heptahydrate, FeSO_4_ 7H_2_O, in a range from 1 mM to 0.1 mM were prepared to obtain the calibration curve. The reaction was carried out for each sample in a final volume of 272 µL in wells. The reaction mixture was incubated at 37 °C for 30 min in dark conditions. The absorbance of the blank, consisting of FRAP alone and monitored spectrophotometrically at the wavelength of 593 nm, was subtracted from the absorbance of the FRAP with the sample to determine the FRAP value for each sample. The FRAP values were determined using the FeSO_4_ 7H_2_O calibration curve [22] and expressed as mmol Fe^2+^/g of hydroalcoholic extract. Trolox was used as the standard reference.

### 2.7. Anti-Enzymatic Activities

#### Anti-Acetylcholinesterase (AChE), Anti-Butyrylcholinesterase (BChE), and Anti-α-Amylase Activities

The AChE and BChE inhibitory assays were performed according to a described spectrophotometric method [23] with some modifications [24]. The α-amylase assay was carried out following Bernfeld’s protocol [25] with some modifications. The color reagent was the dinitrosalicylic acid (DNSA), and it was prepared using 96 mM 3,5-DNSA in ultrapure water (20 mL), 5.3 M potassium sodium tartrate in 2 M NaOH (8 mL), and warm ultrapure water (12 mL). Alpha-amylase was dissolved in ultrapure water (1U). One mg of rosemary extracts was dissolved in 1 mL of water (1 mg/mL) to prepare the stock solution. An amount of 100 µL of different concentrations of hydroalcoholic extracts was mixed with 100 µL of alpha-amylase solution and 200 µL of 20 mM phosphate buffer (pH 6.9, containing 6.7 mM NaCl), and the mixture was incubated for 10 min at 37 °C. After pre-incubation, a starch solution (1% *w*/*v*) (180 µL) was added, and the reaction was incubated at 37 °C for 10 min. Then, a DNSA reagent (180 µL) was added, incubated at 100 °C for 10 min. Finally, the reaction mixture was diluted with distilled water (600 µL). Absorbance was measured at 540 nm using a Thermo Scientific Multiskan GO spectrophotometer (Thermo Fischer Scientific, Vantaa, Finland). The negative control was obtained by replacing the extract with 100 µL of deionized water; no enzyme solution was put in the blank sample. Acarbose was used as the positive control. The result was expressed as an IC_50_ value, which is the concentration of extract necessary to reduce the activity of α-amylase by 50%.

### 2.8. Cell Culture

J774A.1 murine monocyte/macrophage cells were purchased from American Type Culture Collection (ATCC), cultured in Dulbecco’s modified Eagle’s medium (DMEM) supplemented with 10% fetal bovine serum. The J774A.1 cells were incubated at 37 °C in a humidified incubator containing 5% CO_2_.

### 2.9. MTT Assay

J774A.1 cell viability was tested using the 3-[4,5-dimethyltiazol2yl]-2,5 diphenyl tetrazolium bromide (MTT) assay. The cells were plated (3 × 10^3^ cells/well) in a 96 multi-well. The next day, the J774A.1 cells were treated with increasing concentrations (0.01–100 µg/mL) of the two extracts. At 24 h from the treatment, 25 µL of MTT (Sigma-Aldrich, Milan, Italy) (5 mg/mL in saline) was added in each well and the plate was incubated at 37 °C for 3 h. The produced blue formazan crystals were solubilized with a DMSO solution. The absorbance of each well was measured at 545 nm with a microplate spectrophotometer reader (Multiskan FC, Thermo Scientific™, Waltham, MA, USA).

### 2.10. Quantitative Real-Time PCR

The J774.1 macrophages (5 × 10^5^ cells/well) were treated with WRO and CRO (10 µg/mL) for 1 h before the stimulation with LPS/IFN-γ. After 6 h of incubation, total RNA was extracted using TRI-Reagent (Sigma-Aldrich, Milan, Italy), according to the manufacturer’s instructions. Subsequently, cDNA was obtained via reverse transcription with iScript Reverse Transcription Supermix (Bio-Rad, Segrate, Italy). Quantitative real-time PCR (RT-PCR) was performed using a CFX384 real-time PCR detection system (Bio-Rad, Segrate, Italy). mRNA expression was quantified using specific primers for mouse Nos2, which are listed below, with an SYBR Green master mix kit (Bio-Rad, Segrate, Italy). Relative gene expression was obtained by normalizing the Ct values of each experimental group against the β-actin transcript level, using the 2-ΔCt formula.

Nos2: 5′-CGAAACGCTTCACTTCCAA-3′; 5′-TGAGCCTATATTGCTGTGGCT-3′

IL-6: 5′-CGGAGAGGAGACTTCACAGAG-3′; 5′-ATTTCCACGATTTCCCAGAG-3′

TNF-α: 5′-CAGTAGACAGAAGAGCGTGGT-3′; 5′-AGGCACTCCCCCAAAAGA-3′

PTGS2: 5′-CCTGCTTGAGTATGTCGCAC-3′; 5′-TACCCTCCTCACATCCCTGA-3′

### 2.11. Intracellular ROS and Calcium Concentration Measurement

Reactive oxygen species (ROS) and calcium fluxes were measured by using the fluorescence probe 2′,7′-dichlorofluorescein-diacetate (DCF-DA) and FLUO3-AM. First, the J774.A1 cells were seeded into 100 mm dishes (2 × 10^6^ cells/well); then, the cells were treated with WRO and CRO (10 µg/mL) for 1 h and then stimulated with LPS (100 ng/mL) IFN-γ (20 ng/mL) for 24 h. At the end of the incubation, the cells were stained with DCF-DHA (10 μM) and FLUO3-AM (4 μM) for 30 min at 37 °C. Fluorescence generation was measured by FACS (BriCyte E6, Mindray, Shenzhen, China) and analyzed with FlowJo software (Tree Star V.10; Carrboro, NC, USA).

### 2.12. Quantification of Nitrite in Cell Culture Supernatants

The J774A.1 murine macrophages were plated in a 48 multi-well (2 × 10^5^ cells/well) and treated with WRO and CRO (10 µg/mL). After 60 min, the cells were polarized into the M1 phenotype via stimulation with LPS from *Escherichia coli* (O111:B4, Sigma-Aldrich) (100 ng/mL) and IFN-γ (20 ng/mL) (Miltenyi Biotec, Bologna, Italy). Quantitative real-time PCR (RT-PCR) was performed using CFX384 real-time PCR for 24 h. The day after, the medium was collected and the nitrite concentration was evaluated via a Griess reaction. Griess’ reagent (1% sulfanilamide in 5% phosphoric acid and 0.1% N-1-naphthylethylenediamine dihydrochloride in double-distilled water) was mixed with the culture medium in a one-to-one ratio. The multi-well was incubated for 10 min at room temperature, and then, the absorbance was measured at 550 nm using a microplate photometer reader (Multiskan FC, Thermo Scientific™, Waltham, MA, USA). The absorbance values were interpolated with those of the standard curve generated via a serial dilution of sodium nitrite (160 µM–1.25 µM).

### 2.13. Flow Cytometry

The J774A.1 cells were plated in a 24 multi-well and treated with WRO and CRO (10 μg/mL) for 1 h before the stimulation with LPS/IFN-γ for 24 h. The cells were incubated with a fixable viability dye (BioLegend, San Diego, CA, USA). Quantitative real-time PCR (RT-PCR) was performed using CFX384 real-time PCR for the live/dead gate and then fixed and permeabilized with Intracellular Fixation & Permeabilization Buffer (Thermo Fisher Scientific Waltham, MA, USA). After 30 min incubation, the cells were stained with anti-NOS2 (PE-Cy7) (Thermo Fisher Scientific Waltham, MA, USA) for 30 min at room temperature. For live vs. dead status, the J774 cells were treated with WRO and CRO for 24 h, then were labeled with the Zombie Green Fixable Viability Kit (BioLegend, San Diego, CA, USA), and washed as stated by the manufacturer’s instructions. The samples were acquired with a BriCyte E6 flow cytometer (Mindray, Medical Italy S.r.l, Milan, Italy) and analyzed with FlowJo software (Tree Star V.10; Carrboro, NC, USA).

### 2.14. Western Blot Analysis

The J774.A1 cells (2 × 10^6^ cells/well) were plated into dish and treated with WRO and CRO (10 µg/mL) for 1 h before the stimulation with LPS/IFN-γ for an additional 30 min. Nuclear, cytosolic, and total extracts were prepared as previously described [26]. Protein concentration was measured via the Bradford method (Bio-Rad). Amounts of 40 µg of the cytosolic and total extracts and 20 µg of nuclear extract were separated via sodium dodecyl-sulfate polyacrylamide gel electrophoresis (SDS-PAGE) and transferred to polyvinylidene difluoride (PVDF) filter membranes using a Trans-Blot Turbo Transfer Starter System (Bio-Rad). The membranes were blocked with 5% low-fat milk in PBS with 0.1% Tween 20 (PBST) at room temperature for 2 h and, then, were incubated with the following primary antibodies: NF-κB p65 XP (#8242; Cell Signaling, Milan, Italy), IκB-α (#9242, Cell Signaling, Milan, Italy), Cox2 (610204; BD Bioscience, Milan Italy), glyceraldehyde 3-phosphate dehydrogenase (GAPDH) (#2118; Cell Signaling, Milan, Italy), and α-TUBULIN (#3873; Cell Signaling, Milan, Italy) overnight at 4 °C. After three washes with PBST, the membranes were incubated with anti-mouse (Santa Cruz Biotechnology, Heidelberg, Germany) or anti-rabbit (Jackson ImmunoResearch, Milan, Italy) secondary antibody, and horseradish peroxidase (HRP) conjugate for 2 h at room temperature. The membranes were developed with the ChemiDoc™ MP Imaging System (Bio-Rad, Milan, Italy) via the ECL chemiluminescence method.

### 2.15. Prostaglandin E2 Assay

PGE2 concentrations was evaluated in cell culture supernatants obtained after 24 h of treatment with the extracts (10 µg/mL) and stimulation with LPS/IFN-γ. A prostaglandin E2 EIA kit (Cayman Chemicals, Ann Arbor, MI, USA) was used according to the manufacturer’s instruction.

### 2.16. NF-kB Activity Assay

NF-κB activity was measured using a NF-κB p65 Transcription Factor Assay Kit (CAS. #10007889, Cayman Chemical, Shanghai, China) according to the manufacturer’s protocol.

### 2.17. Statistical Analysis

All assays were carried out in triplicate. The statistical analysis was conducted using one-way ANOVA followed by Tukey’s multiple comparisons test at the significance level of *p* < 0.05 using GraphPad Prism 6.0 and MATLAB 2021a software.

## 3. Results

### 3.1. Chemical Analisys of Cultivated and Wild Extracts

#### 3.1.1. Chemical Composition

**Figure 1:**
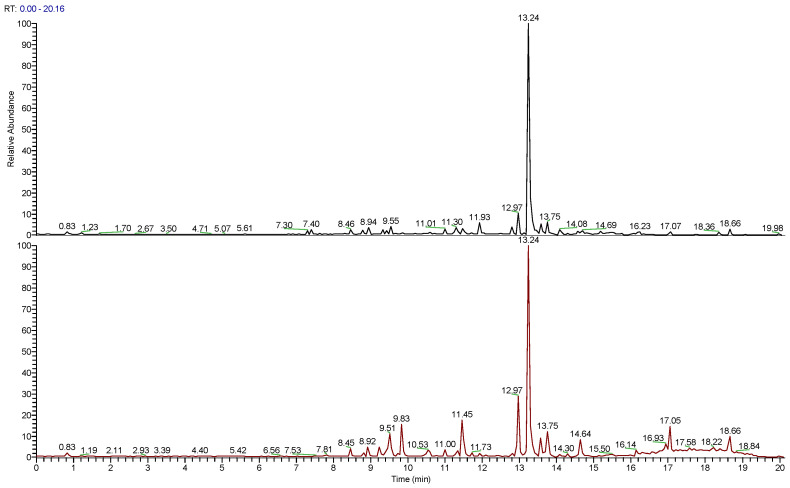
Full scan LC-MS chromatograms (positive-ion HRESIMS) of hydroalcoholic extracts of wild (WRO, upper) and cultivated *R. officinalis* (CRO, lower).

The LC-HRESIMS/MS (Figure 1) analyses of the studied extracts led to the separation and annotation of the most constituents; overall, 14 components (Table 1) were identified as belonging to two representative classes of constituents: flavonoids (peaks 1, 2, 4–9) and terpenoids (peaks 10–14). Rosmarinic acid (peak **3**) at *t*_R_ 8.94 min was the only identified hydroxycinnamic derivative.

The flavonoid profile of the rosemary extracts consisted of a large group of flavones (2, 4, 5, 7, 9, and 10) with different glycosylation and methoxylation degrees, of two flavonol derivatives (**1** and **6**), and of one flavanone (**8**). Compound **1** gave a [M+H]^+^ ion at *m*/*z* 479.1167, attributed to isorhamnetin-3-*O*-glucoside. The fragment peaks observed in the MS spectra of both the cultivated and wild types displayed the loss of a sugar moiety (162 amu), resulting in fragment ions at *m*/*z* 317, corresponding to a protonated molecular ion of isorhamnetin. This assignment was confirmed with the further fragmentation of the ion at *m*/*z* 317 eluted as peak **6** at *t*_R_ 9.83 min, which produced ions at *m*/*z* 302 and *m*/*z* 271, corresponding to a following loss of the methyl and methoxy groups, as reported in the literature [27]. Thus, peaks **1** and **6** have been assigned, respectively, as isorhamnetin-3-*O*-glucoside and its aglycone isorhamnetin.

Regarding the flavone profile of the extracts, the fragment peaks for compound **2** with [M+H]^+^ at *m*/*z* 463.1220 showed the loss of glucose and of a methyl group, respectively, allowing the assignment as homoplantaginin, previously found in rosemary extracts [28,29].

On the other hand, the annotation of the two luteolin-3′-*O*-β-D-glucuronide isomers eluted at 9.49 and 9.55 min, with the same [M+H]^+^ ion at *m*/*z* 505.0957, required the integration of an LC-MS analysis with the chromatographic purification and NMR spectroscopic investigation. Peaks **4** and **5** could be observed in the chromatograms of both extracts. In rosemary extracts, the presence of multiple peaks for luteolin-3′-*O*-glucuronide was reported [30]: the first isomer (peak 5) could be attributed to luteolin-3’-*O*-(2″-*O*-acetyl)-β-D-glucuronide due to its fragmentation pattern (fragment ions at *m*/*z* 285 and 399 corresponding to [M+H–C_8_H_10_O_7_]^+^ and [M+H–C_3_H_4_O_4_]^+^), and the second one (peak 4) could be identified based on the same MS^2^ data as the isomers luteolin-3′-*O*-(3″-*O*-acetyl)-β-D-glucuronide or luteolin-3′-*O*-(4″-*O*-acetyl)-β-D-glucuronide. The existence of multiple positional isomers of this compound in rosemary cannot be distinguished due the limitation of this powerful technique (LC-MS). Since this compound could not be identified based on the available data, an unambiguous assignment was then obtained via isolation and NMR spectroscopic analysis of the purified compound. To this goal, both rosemary extracts (wild and cultivated) were subjected to a reverse-phase HPLC purification and spectroscopic analysis. A part of the both rosemary extracts (15 mg) was separated via semipreparative HPLC-UV using a C18 column with an elution gradient from MeCN/H_2_O 95:5 to MeCN/H_2_O 5:95 in 40 min. Compound **4** (0.5 mg, 3%) was eluted at *t*_R_ 23.6 min from both extracts. The only report about the isolation and structure elucidation of luteolin-3′-*O*-glucuronide isomers was by Okamura et al., 1994 [31], where the assignments of not all the NMR resonances were reported using DMSO-*d_6_* as a solvent. However, in the laboratory experience, the best NMR spectra resolution was obtained using CD_3_OD as a solvent, prompting us to reinvestigate the structure of **4** in detail using 2D NMR experiments.

A pure compound **4** was isolated as an amorphous solid with molecular formula C_23_H_21_O_13_ (by HRESIMS). The ^1^H NMR spectrum of **4** (CD_3_OD) clearly evidenced the presence of the luteolin aglycone, highlighted by the characteristic resonances of ring A (*δ*_H_ 6.18, H-6 and 6.61, H-8, both bs), a methine at *δ*_H_ 6.63, H-3; of a conjugated double bond Δ^2,3^; and of the two doublets (*δ*_H_ 6.98, H-5′ and 7.60. H-6′) and one singlet (*δ*_H_ 8.08, H-2′) of ring B. The midfield region of the ^1^H NMR spectrum also contained signals between δ_H_ 3.73 and 5.11 related to the sugar moiety. The inspection of 2D NMR HSQC and COSY cross-peaks, of proton−proton coupling constants and ^13^C-NMR, allowed us to build up the sugar spin systems, assigning it as a β-D-glucuronic acid residue. Cross-peaks of the 2D NMR HMBC spectrum supported the structural assignment of these moieties but also provided key evidence to link the glucuronic acid at C-3′ of luteolin and to definitely assign the acetylation position at C-3″. Thus, compound **4** was assigned as luteolin-3′-*O*-(3″-*O*-acetyl)-β-D-glucuronide. The pure compound **4** displayed the same retention time of peak 4, thus confirming the attribution. Compound 7, eluting at 10.53 min in cultivated rosemary, showed fragments at *m*/*z* 285.03, 255.03, 227.03, and 209.12. These fragments, resulting from consecutive losses of methyl and CO moieties, are characteristic of the diosmetin, a flavone containing a methoxy group at the 4′ position of the B-ring.

The base peak at *m*/*z* 285.0754 [M+H]^+^ observed in both the chromatograms (*t*_R_ 11.93) has been attributed to the presence of the flavonoid genkwanin corresponding to the 7-*O*-methylapigenin.

The last flavonoid, compound **10** eluted at 12.9 min, gave the protonated molecular ions at *m*/*z* 329.1024 for which molecular formula C_18_H_16_O_6_ was generated. The fragmentation of this compound was in agreement with salvigenin, a polymethoxylated flavone, fragmentation pattern previously reported [32], where it primarily loses a methyl group to form [M+H-CH_3_]^+^, with ions at *m*/*z* 314.0802; further dehydrates to [M+H-CH_3_-H_2_O]^+^, with ions at *m*/*z* 296; and then loses a carbonyl group [M+CH_3_–H_2_O–CO]^+^ at *m*/*z* 268. Finally, rosmadial (*m*/*z* 345.2049 [M+H]^+^), carnosol (*m*/*z* 331.1898 [M+H]^+^), carnosic acid (*m*/*z* 333.2052 [M+H]^+^), and 12-methoxy carnosic acid (*m*/*z* 345.21 [M+H]^+^) were identified as the major diterpenoids.

#### 3.1.2. Quantitative Analysis of Major Phenolic Compounds

The quantitative profiling of the hydroalcoholic extracts of *R. officinalis* was performed via a high HPLC-UV analysis using a UV detection wavelength at 280 nm.

Since salvigenin (**10**) is present in both the WRO and CRO extracts, it was used as an internal standard, allowing for the relative quantification of all the other UV-detected compounds. The coefficient determination (R^2^) of the calibration curve had good fitness (0.9975–0.99874), indicating the reliability of the method. As shown in Table 2, the major component of both the WRO and CRO extracts is rosmarinic acid (**3**), followed by salvigenin (**10**) and isorhamnetin (**6**) in CRO, and only by salvigenin in WRO, while the other components noted by deterplicating the LC-MS^2^ profiling resulted in being present only in trace amounts.

#### 3.1.3. Total Phenolic and Flavonoid Content

The quantification of total phenolic content was evaluated via the Folin–Ciocalteu colorimetric assay. The amount was expressed as mg of gallic acid equivalents per gram of extract. As report in Table 3, the total phenolic content was 129.36 ± 9.23 mg GAE/g and 175.64 ± 42.61 mg GAE/g in WRO and CRO, respectively. The quantification of total flavonoid content was studied via an aluminum chloride colorimetric assay. The amount was expressed as milligram of quercetin equivalent per gram of extract. The total flavonoid content was 61.67 ± 2.33 mg QE/g and 49.47 ± 6.35 mg QE/g in WRO and CRO, respectively (Table 3).

### 3.2. Antioxidant Activity: DPPH and FRAP Assay

The antioxidant activity was determined using two colorimetric and spectrophotometric assays: DPPH and FRAP. The results in Table 3 show that the amount of extract needed to reduce the absorbance of DPPH by 50% is 17.58 µg/mL and 14.93 µg/mL for WRO and CRO, respectively. In the FRAP assay, the WRO and CRO presents 2.25 and 3.66 mmol Fe^2+^ equivalents/g extract, respectively.

### 3.3. Enzymatic Activities

#### Anti-Acetylcolinesterase (AChE), Anti-Butyrylcolinesterase (BChE), and Anti-α-Amylase Activities

The anticholinesterases activities were evaluated using Ellman’s assay. As reported in Table 4, the IC_50_ values for AChE are 96.39 µg/mL and 216.50 µg/mL for WRO and CRO, respectively. The values of IC_50_ for BChE were 243.35 µg/mL and 257.20 µg/mL for WRO and CRO, respectively.

The hydroalcoholic extracts of *Rosmarinus officinalis* have been studied for their possible inhibitory activity on the α-amylase enzyme. As reported in Table 4, the IC_50_ values are 40.52 µg/mL and 52.68 µg/mL for WRO and CRO, respectively.

### 3.4. Effect of Rosmarinus Officinalis Extracts on Macrophage Cell Viability

**Figure 2:**
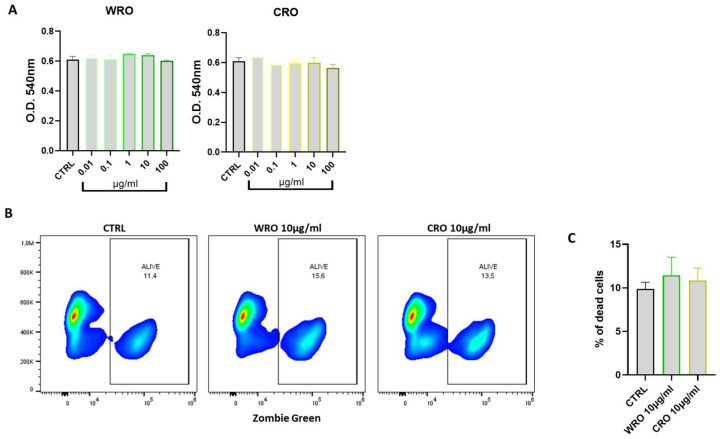
(**A**) J774 A.1 cells were treated with increasing concentrations of WRO and CRO. Cytotoxicity was evaluated via MTT assay 24 h after treatment. (**B**) Representative plot and (**C**) frequency of dead cells after treatment for 24 h with both CRO and WRO at 10 μg/mL.

The J774A.1 murine macrophages were used to investigate the anti-inflammatory and antioxidant effects of extracts. First, the cytotoxic effect of both WRO and CRO extracts was evaluated. The J774A.1 macrophages were treated with increasing concentrations of WRO and CRO (from 0.01 to 100 µg/mL) for 24 h, and cell viability was assessed via the MTT assay. As shown in Figure 2, both extracts did not affect the viability of the J774A1 macrophages at all tested concentrations. Therefore, the concentration of 10 µg/mL was selected for characterizing the anti-inflammatory and antioxidant effects. In addition, a flow cytometry analysis, with the Zombie green probe in order to differentiate live vs. dead cells status, was performed. As shown in Figure 2B,C, both extracts did not increase the percentage of dead cells, confirming the non-cytotoxic effect of both WRO and CRO at 10 µg/mL.

### 3.5. WRO and CRO Extracts Inhibited the Production of TNF- and IL-6 by Stimulated Macrophages

**Figure 3:**
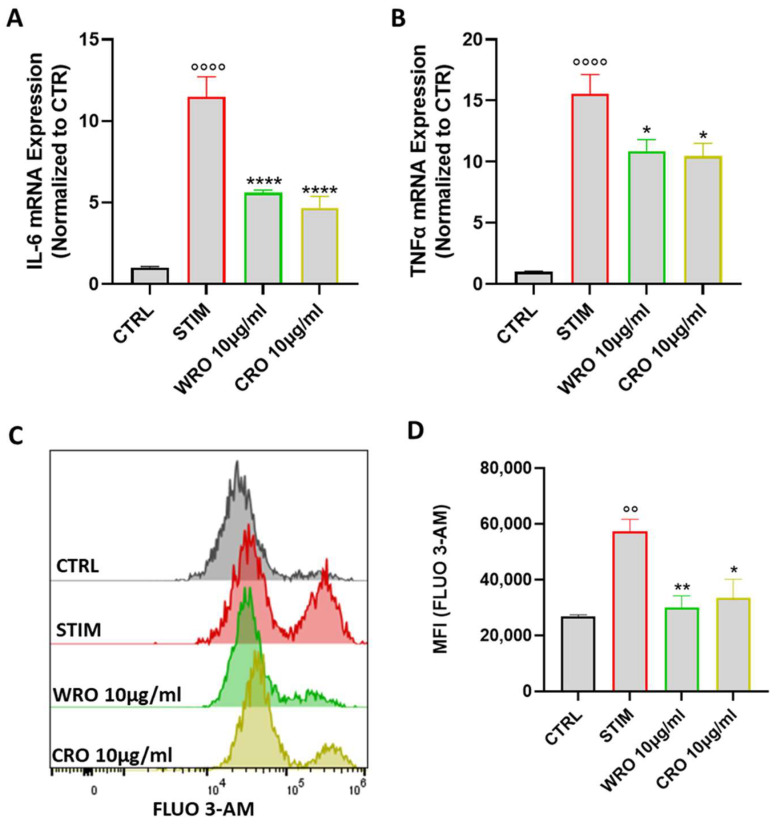
(**A**,**B**) J774A.1 cells were treated with extracts (10 μg/mL) for 1 h before stimulation with LPS (100 ng/mL) and INF-γ (20 ng/mL) for 6 h. Subsequently, mRNA levels of the pro-inflammatory cytokines IL-6 (**A**) and TNF-α (**B**) were determined. (**C**) Representative example of flow cytometry analysis of FLUO3-AM staining. (**D**) FLUO3-AM quantification in terms of mean fluorescence intensity (MFI). Values were expressed as mean ± SEM from at least three independent experiments. °° *p* < 0.01, °°°° *p* < 0.0001 indicate a significant effect of LPS/INFγ compared with unstimulated cells (CTRL); * *p* < 0.05, ** *p* < 0.01, **** *p* < 0.0001 indicated a significant effect of CRO and WRO compared with LPS/INF-γ-stimulated cells.

In order to evaluate the capability of the extracts to modulate the function of macrophages, the pro-inflammatory M1 phenotype in J774 macrophages was induced through stimulation with LPS (100 ng/mL) and IFN-γ (20 ng/mL). In particular, J774 macrophages were pretreated for one hour with WRO and CRO prior to stimulation with LPS and IFN-γ for 6 h. Next, the mRNA expression levels of TNF-α and IL-6, the main cytokines secreted by activated macrophages [33], were evaluated. As shown in Figure 3A,B, stimulation with LPS and INF-γ markedly increased the mRNA expression levels of both IL-6 and TNF-α. Conversely, pre-treatment with WRO and CRO significantly reduced the expression of these two genes. The increase in calcium plays a notable function in LPS-induced secretion of cytokines by macrophages [34]. Thus, whether the extract-mediated IL-6 and TNF-α reduction was correlated with the modulation of calcium fluxes in the J774 macrophages was evaluated using the fluorescent probe Fluo 3-AM [35]. In line with the qPCR data, the pretreatment with extracts significantly reduced the calcium concentration compared with macrophages stimulated with LPS and INF-γ for 24 h (Figure 3C,D).

### 3.6. WRO and CRO Extracts Modulated the Nitric Oxide (NO) Pathway in Activated Macrophages

**Figure 4:**
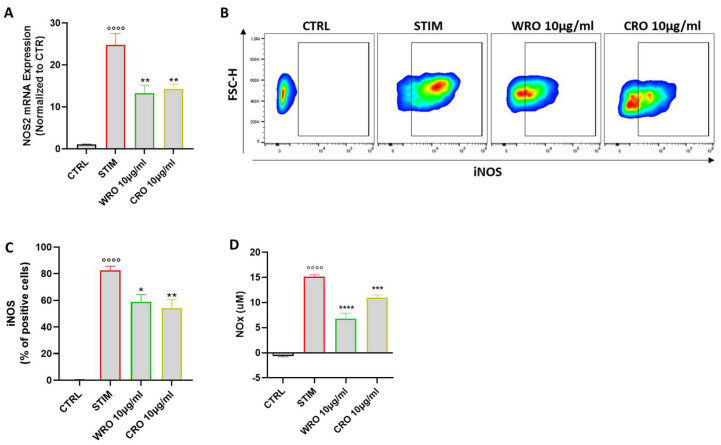
(**A**) Relative mRNA levels of NOS2 in J774 macrophages were determined using qPCR analysis after 6 h. (**B**) Representative plot and (**C**) frequency of intracellular NOS2 expression in J774 macrophages evaluated via flow cytometry after 24 h. (**D**) Levels of NO measured in the cell culture medium of J774 cells after 24 h via the Greiss reaction. Values were expressed as mean ± SEM from at least three independent experiments. °°°° *p* < 0.0001 indicates a significant effect of LPS/INFγ compared with unstimulated cells (CTRL); * *p* < 0.05, ** *p* < 0.01, *** *p* < 0.001, **** *p* < 0.0001 indicated a significant effect of WRO and CRO compared with LPS/INF-γ-stimulated cells.

To better characterize the anti-inflammatory effect of WRO and CRO, their ability to modulate the NO pathway, one of the major players involved in macrophage activation [36], was also investigated. First, the expression of NOS2 (also referred to as NOS2), the inducible form of the nitric oxide synthase enzyme expressed in M1 macrophages [37], was taken in consideration. As shown in Figure 4A, the NOS2 expression in J774 cells was significantly reduced following treatment with the WRO and CRO extracts compared with J774 stimulated with LPS and INFγ. These data were further confirmed at the protein level, as demonstrated using the flow cytometry analysis of NOS2 protein expression level (Figure 4B,C). Finally, the effective secretion of NO by performing the Griess assay was also evaluated. In line with the findings above, pretreatment with the *Rosmarinus* extracts significantly suppressed NO secretion compared with the LPS/INFγ stimulated macrophages (Figure 4D).

### 3.7. WRO and CRO Exert Antiinflammatory Effect by Inhibiting NF-κB Activation

**Figure 5:**
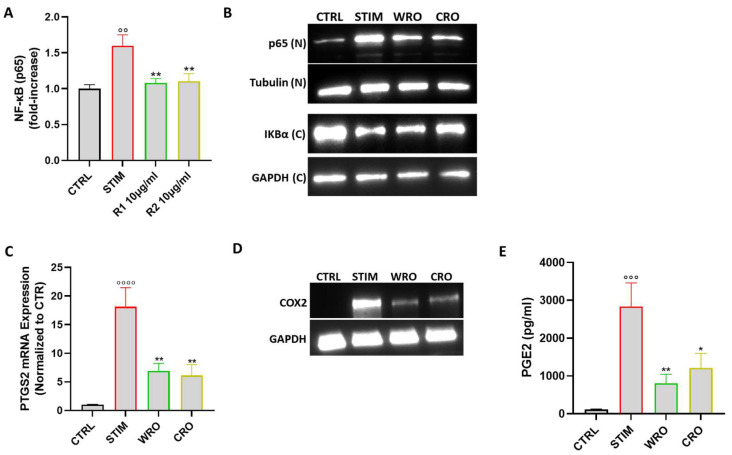
(**A**) Nuclear extracts were prepared using a nuclear extract kit. NF-κB-P65 activity was measured using an ELISA kit. (**B**) Representative images of p65 and IkB-α proteins detected via Western blot analysis, respectively, in cytosolic (**C**) and in nuclear (N) extract after 1 h of pre-treatment with 10 µg/mL of WRO and CRO and 30 min of LPS/IFN-γ stimulation. α-tubulin and GAPDH were used as an internal control. (**C**) mRNA levels of PTGS2 after treatment with WRO and CRO (10 μg/mL) for 1 h and stimulation with LPS (100 ng/mL) and INF-γ (20 ng/mL) for 6 h. (**D**) Representative images of COX-2 proteins detected via Western blot analysis in total extract. GAPDH was used as an internal control. (**E**) PGE2 concentrations in J774 cell culture supernatants after 24 h of treatment with the extracts (10 µg/mL) and stimulation with LPS/IFN-γ. Values were expressed as mean ± SEM from at least three independent experiments. °° *p* < 0.01, °°° *p* < 0.001, °°°° *p* < 0.0001 indicate a significant effect of LPS/INFγ compared with unstimulated cells (CTRL); * *p* < 0.05, ** *p* < 0.01, indicate a significant effect of CRO and WRO compared with LPS/INF-γ-stimulated cells.

It is known that NO overproduction in LPS-stimulated macrophages is linked to the activation of inducible nuclear factors, including NF-kB [38]. Therefore, the anti-inflammatory effect of WRO and CRO by evaluating the activation of NF-kB was further examined. In particular, the nuclear translocation of NF-κB p65 subunit via both an NF-kB activity assay and a Western blot analysis was evaluated: upon stimulation with LPS/INFγ, p65 nuclear levels were markedly increased, whereas pre-treatment with both WRO and CRO significantly reduced the p65 expression into the nuclei of macrophages (Figure 5A,B). In addition, the expression of the IκB-α protein, which inhibits the DNA binding of NF-κB subunits by keeping them retained in the cytoplasm, was evaluated. As shown in Figure 5C, pretreatment with WRO and CRO partially restored the cytoplasmatic IκB-α levels in the J774 macrophages. The cyclooxygenase 2 (COX-2) enzyme is largely regulated by NF-κB since COX-2 binding sites are in the promoter region of the NF-κB transcription factor complex [39]. Thus, whether the extracts were able to modulate the expression of COX-2 and the production of PGE2, the major prostanoid produced via COX-2 activity and involved in inflammatory processes, was also investigated. The qPCR analysis demonstrated that the COX-2 mRNA expression levels were significantly upregulated in the J774 cells stimulated with LPS/INFγ, whereas pretreatment with WRO and CRO restored the expression of COX-2 to almost those at the basal levels. This result has also been confirmed at the protein levels via a Western blot analysis. In line with this finding, the production of PGE2 was significantly reduced in the supernatant of both the WRO- and CRO-treated J774 cells. Taken together, these results unveil the potential mechanism of action underlying the anti-inflammatory activity of both the WRO and CRO extracts explicated through the inhibition of NF-kB and its downstream mediator COX-2.

### 3.8. WRO and CRO Extracts Reduced ROS Production and Impaired Mitochondrial Function

**Figure 6:**
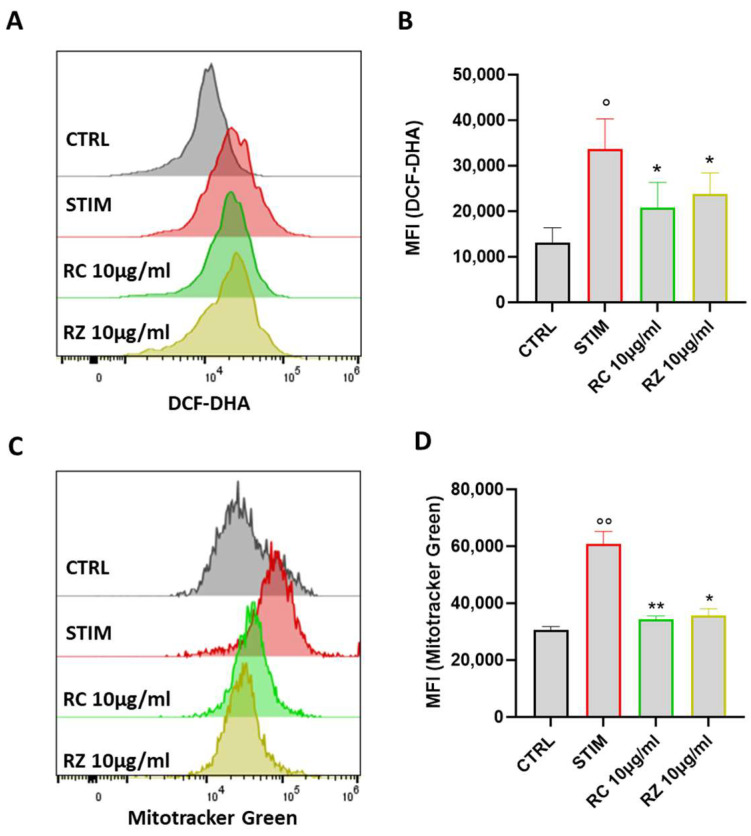
(**A**) Representative example of flow cytometry analysis of DCF-DHA probe. (**B**) DCF-DHA quantification in terms of mean fluorescence intensity (MFI). (**C**) Representative examples of flow cytometric analysis of MitoTracker Green (**C**) with its quantification in terms of mean fluorescence intensity (MFI) (**D**). Values were express as mean ± SEM from three independent experiments. ° *p* < 0.05, °° *p* < 0.01, indicate significant effect of LPS/INFγ-stimulated cells compared with unstimulated cells (CTRL); * *p* < 0.05, ** *p* < 0.01 indicated significant effect of WRO and CRO extracts compared with stimulated cells.

To test whether the WRO and CRO extracts exerted an antioxidant effect, the generation of intracellular reactive oxygen species (ROS) in M1-polarized macrophages was studied. Exposure of cells to INFγ/LPS for 24 h induced a significant increase in ROS generation, as assessed using the fluoroprobe DCF-DHA, whereas pre-treatment with WRO and CRO (10 μg/mL) reduced ROS production (Figure 6A,B). Moreover, since mitochondria are the major sources of ROS [40], the effects of WRO and CRO on mitochondrial mass were considered. As shown in Figure 6C,D, the uptake of the Mito-Tracker Green dye was significantly reduced in M1-polarized J774 macrophages compared with cells stimulated with INFγ and LPS.

## 4. Discussion

In vitro cultivated plants can be used to maintain the populations of the main medicinal plant species that could be used for the preparation of herbal remedies: for this reason, it is important to track the differences and to compare the biological activities of extracts from cultivated plants and those grown in natural habitats [41].

In the present study, a total of 14 compounds were detected in hydroalcoholic cultivated and wild rosemary extracts using an LC-MS/MS analysis. The compounds were purified and isolated via HPLC and their structures were identified via NMR experiments to obtain in-depth knowledge of the constituents of the rosemary extract to unambiguously attribute the peaks of the LC-MS profile. This in-depth analysis was necessary especially for the assignment of peak 4, since LC-MS could not provide an unambiguous distinction between 3″-*O*-acetyl and 4″-*O*-acetyl isomers of luteolin-3′-*O*-β-D-glucuronide, since these two compounds show identical fragmentation patterns. Therefore, a chromatographic purification was performed, and since a better resolution of NMR spectra was obtained in CD_3_OD, a reinvestigation of the luteolin-3′-*O*-(3″-*O*-acetyl)-β-D-glucuronide structure was conducted, proving the unambiguous presence of this isomer in rosemary extracts. The findings show that cultivation did not greatly affect rosemary extract compositions.

In this study, for the first time, the capability of rosemary extract and its chemical constituents to modulate the macrophage-mediated inflammatory process was shown. In particular, the studied extracts thwarted the production of mitochondria-mediated ROS and suppressed the inflammatory process through a reduction in the major pro-inflammatory cytokines and nitric oxide (NO).

The total phenolic contents in WRO and CRO confirmed studies in the literature [13,42,43]; the total flavonoid contents in the WRO and CRO were higher than those in previously reported studies [44].

The antioxidant power of the extracts, evaluated via DPPH (Table 2), confirmed the data reported by Conforti et al. [45], who demonstrated a significant radical scavenging activity for the rosemary hydroalcoholic extract and asserted that several scientific articles correlated the antioxidant activities of rosemary extracts with the presence of specific polyphenol components [45]. Also, Ali and coworkers [46] reported on the antioxidant activity of a rosemary methanolic extract being probably dependent on its phenolic and flavonoid content. In the literature [5], a greater quantity of polyphenols than half of the compounds have the catechol group, which is associated with a greater antioxidant power: the phytochemical analysis of the plant extracts showed the existence of three natural products classes: terpenes, polyphenols, and flavonoids. In accordance with this study, the antioxidant activity was linked to the presence of phenols and flavonoids in the plant extracts, complex mixtures of several classes of compounds [5,47,48], and is probably the product of additive, synergistic, and/or antagonistic effects. Ho and coworkers [47] reported very good radical scavenging activities of flavonoid compounds, such as luteolin 7-*O*-glucopyranoside. Carnosic acid and the total amount of phenolic diterpenes were considered major contributors to rosemary antioxidant activity [49,50]. According to a FRAP assay, the extracts had an interesting content of Fe^2+^, also confirmed by Kabubli and coworkers [51]. In the literature, hydroalcoholic extracts of *Rosmarinus officinalis* showed a lower content of ferrous ion than the tested samples [52,53]. Compared with the data reported here, it appears that the domestication did not greatly affect the antioxidant property of the studied extracts, since the values did not differ much between the wild and cultivated plants.

The anticholinesterases activities were conducted on the AChE and BChE enzymes, which are responsible for the degradation of acetylcholine. The data showed a greater inhibitory activity on AChE exerted by the WRO extract compared with that of the CRO extract instead, and the inhibitory activity on BChE is similar between the two extracts. Probably, the two extracts act in different ways on the two enzymes because AChE and BChE have different sensitivities to the inhibitors [54]. Furthermore, the different inhibitory activities of the two extracts on AChE could be due to the presence of salvigenin, a compound present in higher quantities in CRO than in WRO and not active on AChE [55]. Furthermore, the compound showed an even stimulating effect on the AChE enzyme, an effect that could be related to the different activity manifested by the two extracts [56].

In the literature, few studies on the anti-acetylcholinesterase activity of rosemary hydroalcoholic extracts have been reported. Mata et al. [57] reported an IC_50_ value of a rosemary ethanolic extract comparable with the value obtained for CRO but higher than the IC_50_ value of WRO. More recently, Ali-Shtayeh and coworkers [58] reported an IC_50_ value of a hydroalcoholic extract of *R. officinalis*, collected in North Palestine, far greater than that of the two tested extracts. No previous studies reported the activity of rosemary extracts on BChE. Kamli and coworkers [9] demonstrated that rosmarinic acid could interact with amino acids of AChE showing anticholinesterase activity.

The inhibitory activity on the α-amylase enzyme of both the WRO and CRO extracts was also evident. In the literature, the activity against α-amylase was linked to polyphenolic compounds: the hydroxyl (-OH) groups of phenolic substances were essential for the inhibitory activity against α-amylase. In fact, the formation of hydrogen bonds between the –OH groups of phenolics and the side chains of amino acids at the active site of α-amylase is probably responsible for the enzyme inhibition [59,60]. No studies on the anti-α-amylase activity of rosemary hydroalcoholic extracts have been reported. Buchholz and Melzig [61] reported that the IC_50_ values of both the methanolic and aqueous extracts of rosemary were higher than the IC_50_ values of WRO and CRO. Ali and coworkers [42] asserted that the potent α-amylase inhibitory activity of rosemary extracts was related to their total phenolic and flavonoid contents, as also confirmed via the results reported here. Several phenolic substances, first of all, flavonoids, are known to be potential antidiabetic molecules exerting a good inhibitory effect of α-amylase and could be used as possible preventive agents in diabetes mellitus disease as part of a dietary strategy. At present, few studies have focused on characterizing the anti-inflammatory and antioxidant effects of *Rosmarinus officinalis* extracts. For instance, it has been demonstrated that rosemary extracts are effective in the treatment of orchitis by exerting an antioxidant effect on murine sperm [62]. Likewise, a polyherbal formula based on *Populus nigra* L. and *R. officinalis* extracts demonstrated a therapeutic potential for diseases based on oxidative stress and inflammation [63]. Differently from data on extracts, many reports are available regarding the anti-inflammatory effect of carnosic acid and carnosol using different in vitro and in vivo models of inflammation [64]. For instance, it has been recently demonstrated that carnosic acid thwarts indomethacin-induced gastric ulceration by reducing oxidative stress and attenuating inflammation in a murine model [65]. However, although carnosic acid and carnosol are known for their beneficial activities, these studies exclude the potential anti-inflammatory effect that may be related to other components of the rosemary extract. In this study, both the WRO and CRO extracts demonstrated promising antioxidant and anti-inflammatory activities using the J774 murine macrophages: WRO and CRO were both able to modulate the function of M1 macrophages by reducing the production of the proinflammatory cytokines IL-6 and TNF-α. In line with other results observed with carnosic acid- and carnosol-treated RAW 264.7 macrophages [66,67], both WRO and CRO reduced the inducible nitric oxide enzyme (NOS2) and the consequent NO production. Moreover, our study demonstrated that both WRO and CRO suppress LPS-mediated p65 activation, the critical transactivation subunit for NF-κB, as well as the activation of COX-2 in J774. Finally, the obtained results showed that both extracts demonstrated a great antioxidant activity by reducing ROS production and modulating mitochondrial function. This effect was also recently reported by Guimarães and coworkers [68] where a rosemary extract was demonstrated to protect liver mitochondria from hepatotoxicity-related oxidative stress [68]. These data support the hypothesis that WRO and CRO extracts impair mitochondrial activity reducing their ability to produce ROS and sustain the inflammatory process, confirming that it is possible and safer to use the cultivated rosemary plants instead of wild ones.

## 5. Conclusions

The present study focused on the qualitative characterization of cultivated and wild *R. officinalis* extracts.

From the chemical point of view, the analysis of the two extracts revealed that the similar compositions were correlated with no significant differences in evaluated biological activities. In fact, functional assays demonstrated that both WRO and CRO exhibited significant scavenging activity against the DPPH radical and a good reducing power according to the FRAP assay. Moreover, the study revealed that both wild and cultivated *R. officinalis* have considerable amounts of phenolic compounds, which are mostly stated as powerful antioxidant. In line with these findings, these in vitro studies displayed that the extracts were able to counteract the proinflammatory function of M1 macrophages by modulating the production of proinflammatory cytokines and ROS. Collectively, the obtained results demonstrated that both WRO and CRO could represent a starting point for further in vivo evaluation with the aim to find a therapeutic strategy for inflammation-based diseases: therefore, *R. officinalis* can be recommended to be cultivated for the food and pharmaceutical industries, considering no significant changes in the studied activities of wild and cultivated plants.

## Figures and Tables

**Table 1:** Chemical composition of hydroalcoholic extracts of wild (WRO) and cultivated (CRO) *R. officinalis.* Compounds were listed in order of LC-MS elution and marked with X when present in the analyzed extracts. All mass peaks are [M+H]^+^ adducts.

No	Family	*t_R_* (min)	Measured *m*/*z*	Molecular Formula	Identification	WRO	CRO
1	flavonoid glycoside	8.45	479.1167	C_22_H_22_O_12_	Isorhamnetin-3-*O*-glucoside	X	X
2	flavonoid glycoside	8.78	463.1220	C_22_H_22_O_11_	Homoplantaginin (Hispidulin-7- Glucoside)	X	X
3	polyphenol	8.94	361.0909	C_18_H_16_O_8_	Rosmarinic acid	X	X
4	flavonoid glycoside	9.43	505.0963	C_23_H_21_O_13_	Luteolin-3′-*O*-(3″-*O*-acetyl)-β-D-glucuronide	X	X
5	flavonoid glycoside	9.55	505.0957	C_23_H_21_O_13_	Luteolin-3′-*O*-(2″-*O*-acetyl)-β-D-glucuronide	X	X
6	flavonoid	9.83	317.0644	C_16_H_12_O_7_	Isorhamnetin		X
7	flavonoid	10.53	301.0699	C_16_H_12_O_6_	Diosmetin		X
8	flavonoid	11.30	315.0853	C_17_H_14_O_6_	Pectolinarigenin	X	X
9	flavonoid	11.93	285.0754	C_16_H_12_O_5_	Genkwanin	X	X
10	terpenoid	12.97	329.1008	C_18_H_16_O_6_	Salvigenin	X	X
11	terpenoid	13.24	331.1898	C_20_H_26_O_4_	Carnosol	X	X
12	terpenoid	13.75	317.2103	C_20_H_28_O_3_	Rosmaridiphenol	X	X
13	terpenoid	14.08	333.2052	C_20_H_28_O_4_	Carnosic acid	X	X
14	terpenoid	14.30	345.2049	C_21_H_28_O_4_	Rosmadial	X	

X indicated the presence of compound in the two extracts.

**Table 2:** Quantification of major phenolic compounds from hydroalcoholic extracts of wild (WRO) and cultivated (CRO) *R. officinalis*.

Compound	Amount mg/g	
	WRO	CRO
**1**	9.03	72.93
**2**	30.21	313.12
**3**	6840.56	6494.65
**4**	1.33	265.35
**5**	0.15	22.49
**6**	0.00	1373.01
**7**	0.00	169.21
**8**	4.06	258.07
**10**	203.74	1128.92
**11**	60.52	38.48
**13**	1.77	46.39

**Table 3:** Total phenolic and flavonoid content and antioxidant activity of WRO and CRO.

	TPC mg GAE ^1^/g Extract (Mean ± SD)^4^	TFC mg QE ^2^/g Extract (Mean ± SD)^4^	DPPH IC_50_^3^(µg/mL) (Mean ± SD)^4^	FRAP mmol Fe^2+^ Equivalents/g Extract (Mean ± SD) ^4^
WRO	129.36 ± 9.23 ^a^	61.67 ± 2.33 ^a^	17.58 ± 0.98 ^a^	2.25 ± 0.55 ^c^
CRO	175.64 ± 42.61 ^a^	49.47 ± 6.35 ^b^	14.93 ± 0.92 ^b^	3.66 ± 0.72 ^b^
Trolox	/	/	3.65 ± 0.08 ^c^	5.48 ± 0.34 ^a^

^1^ GAE = gallic acid equivalents. ^2^ QE = quercetin equivalents. ^3^ IC_50_ = concentration required to reduce the absorbance of DPPH by 50%. ^4^ Mean ± SD = indicates the mean value of the three experiments and the value of the standard deviation. Trolox is used as reference standard in antioxidant assays. Means followed by different letters in the same column indicate that are significantly different at *p* < 0.05, according to a two-way ANOVA followed by Tukey’s post hoc test.

**Table 4:** Anti-acetylcolinesterase (AChE), anti-butyrylcolinesterase (BChE), and anti-α-amylase of WRO and CRO.

	AChE IC_50_^1^ (µg/mL) (Mean ± SD) ^2^	BChE IC_50_^1^ (µg/mL) (Mean ± SD) ^2^	α-Amylase IC_50_^1^ (µg/mL) (Mean ± SD) ^2^
WRO	96.39 ± 3.22 ^b^	243.35 ± 11.25 ^a^	40.52 ± 3.57 ^b^
CRO	216.50 ± 19.90 ^a^	257.20 ± 5.50 ^a^	52.68 ± 4.38 ^a^
Galantamine	0.79 ± 0.01 ^c^	1.97 ± 0.14 ^b^	/
Acarbose	/	/	0.95 ± 0.31 ^c^

^1^ IC_50_ = concentration required to reduce the enzymatic activity by 50%. ^2^ Mean ± SD = indicates the mean value of the three experiments and the value of the standard deviation. Galantamine or acarbose were used as reference standards. Means followed by different letters in the same column indicate that they are significantly different at *p* < 0.05, according to a two-way ANOVA followed by Tukey’s post hoc test.

## Data Availability

The data presented in this study are available upon request from the corresponding author.
